# Multiplexed labeling of cellular proteins with split fluorescent protein tags

**DOI:** 10.1038/s42003-021-01780-4

**Published:** 2021-02-26

**Authors:** Ryo Tamura, Fangchao Jiang, Jin Xie, Daichi Kamiyama

**Affiliations:** 1 Department of Cellular Biology, University of Georgia, Athens, GA USA; 2 Department of Chemistry, University of Georgia, Athens, GA USA

**Keywords:** Fluorescence imaging, Cellular imaging

## Abstract

Self-complementing split fluorescent proteins (split FP_1-10/11_) have become an important labeling tool in live-cell protein imaging. However, current split FP systems to label multiple proteins in single cells have a fundamental limitation in the number of proteins that can be simultaneously labeled. Here, we describe an approach to expand the number of orthogonal split FP systems with spectrally distinct colors. By combining rational design and cycles of directed evolution, we expand the spectral color palette of FP_1-10/11_. We also circularly permutate GFP and synthesize the β-strand 7, 8, or 10 system. These split GFP pairs are not only capable of labeling proteins but are also orthogonal to the current FP_1-10/11_ pairs, offering multiplexed labeling of cellular proteins. Our multiplexing approach, using the new orthogonal split FP systems, demonstrates simultaneous imaging of four distinct proteins in single cells; the resulting images reveal nuclear localization of focal adhesion protein Zyxin.

## Abstract (web summary)

Tamura et al. expand the split fluorescent protein system to multi-colour imaging (six-colours). Their multiplexing approach, using the orthogonal split FP systems, allows simultaneous imaging of four distinct proteins in single cells.

## Introduction

In the self-complementing split GFP system, super-folder GFP is split between β-strands 10 and 11, rendering 214-amino acid and 16-amino acid fragments^1^. The short fragment, GFP_11M3 OPT_, acts as an epitope tag when inserted into a gene of interest^2^. When expressed in the same cell, the GFP_1-10 D7_ and GFP_11M3 OPT_ fragments (hereafter referred to as GFP_1-10/11_) spontaneously interact with each other to form a functional GFP (Supplementary Fig. 1). The GFP_11_ fragment has been used in numerous biological studies^3,4^: targeting nanomaterials in cells^5,6^, forming protein oligomeric structures^2,7^, verifying aggregation processes^8^, and imaging protein localization in living cells^9^. By introducing additional substitutions into the GFP_1-10_ fragment, cyan and yellow spectral variants were previously created and used to visualize localization patterns of cellular proteins^2,10^. The majority of substitutions which lead to the spectral shifts in these variants are located within the large fragments (i.e., CFP_1-10_ and YFP_1-10_). These fragments retain the ability to bind to the identical GFP_11_ fragment, so that reconstitution with GFP_11_ produces a functional cyan or yellow FP.

These self-complementing split GFP variants have already become a powerful and versatile tool for various imaging applications. In particular, endogenously tagged cell lines can be produced by the efficient introduction of the short fragment (GFP_11_) into a genomic locus without perturbing local genomic structure^2,11^. Additionally, we have been able to generate a library of human cells with GFP_11_-tagged endogenous proteins via CRISPR/Cas9-mediated homology-directed repair (HDR), and demonstrate that GFP_11_-tag is compatible with a wide range of cellular proteins such as enzymes, receptors, transport proteins, and structural proteins^12^. However, labeling multiple proteins simultaneously in single cells has been challenging. Multiplexed visualization is tremendously beneficial for simultaneous comparisons of protein dynamics. Recently, great advances have been made in split super-folder Cherry (sfCherry_1-10/11_) as a second, orthogonal split FP system^2,13,14^. The GFP_11_ and sfCherry_11_ fragments allow simultaneous labeling of two different proteins. Although this multicolor approach has expanded the potential of split-FP labeling, it has a bottleneck in multiplexing because of the limited number of available orthogonal split FP systems with different colors.

In this report, we expand the color palette of self-associating split FPs. We have introduced rational mutations into the amino acid sequence of EBFP2 through site-directed mutagenesis and generated two blue-colored split FPs, EBFP2_1-10/11_, and Capri_1-10/11_. We have also engineered self-associating fragments of mRuby3 (mRuby3 is a red-colored FP with a shorter-wavelength than sfCherry)^15^. We have evolved mRuby3_1-10_ by a directed evolution strategy to increase its complementation with mRuby3_11_. Our final optimized construct, split mRuby4, becomes a fusion pair when expressed in human cells. In addition, we propose a new approach to generate more orthogonal split FPs using circularly permutated FP fragments. This approach can potentially overcome multiplexing limitations of split-FP labeling. Finally, as a proof-of-concept experiment, we applied our technique to visualize differential distribution of four proteins in single human cells and found that focal adhesion protein Zyxin sometimes accumulated in the nucleus.

## Results and discussion

### Rationally designed variants of split BFP and CFP

**Fig. 1:**
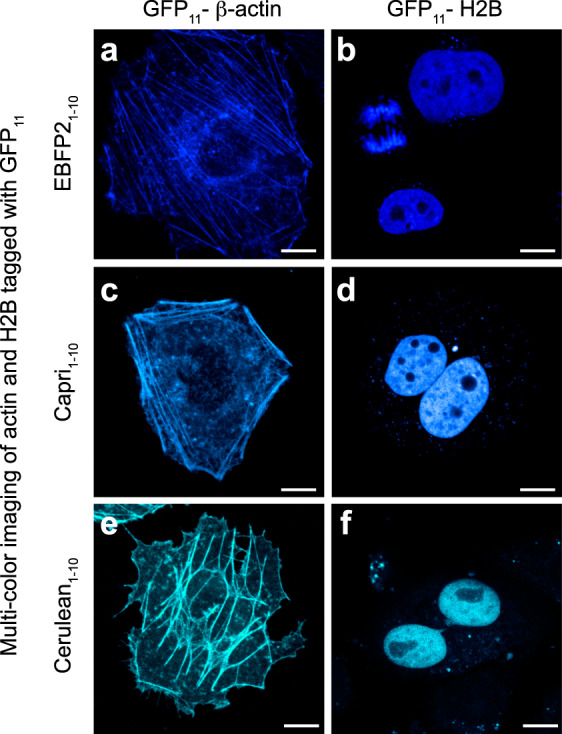
Performance of BFP and CFP_1-10/11_ variants in fusion constructs. **a**–**f** Confocal images of HeLa cells. Cells co-expressing EBFP2_1-10_ (**a**, **b**), Capri_1-10_ (**c**, **d**), and Cerulean_1-10_ (**e**, **f**) with GFP_11_-tagged β-actin or histone 2B.

To expand our color palette of split FPs, we split EBFP2 at the same site as GFP_1-10/11_ (note that EBFP2 is 4-fold brighter and >500-fold more photostable than EBFP^16^). While the short fragment is identical to the amino acid sequence of GFP_11_, six substitutions have been introduced into the large fragment through site-directed mutagenesis (N40I/T106K/E112V/K166T/I167V/S206T; the numbering of amino acids follows that of EBFP2). These substitutions have been previously shown to enhance complementation efficiency of GFP_1-10_ variants^1^. To verify in vivo complementation between the two fragments, we used GFP_11_-tagged β-actin and histone 2B. Co-expressing each one with EBFP2_1-10_ in HeLa cells, we observed blue fluorescence in images of the actin stress fibers and the nucleoplasm (Fig. 1a, b). In some cases (e.g., the actin image), autofluorescence limits the usefulness of this split construct because its overall fluorescent signal is extremely weak. In fact, high autofluorescence background with UV light is often observed in the perinuclear region (Supplementary Fig. 2). To improve its overall brightness, we decided to add six more substitutions to EBFP2_1-10_ (S65T/Q80R/F99S/V128T/M153T/V163A; some of these have previously been characterized to promote the stability and folding rate of GFP^1,17^). This new split FP, termed split Capri for its cyan–blue color, has the same absorption spectrum as split EBFP2 (Supplementary Fig. 3). The emission spectrum, however, is red-shifted from split EBFP2 by 20 nm (*λ*_abs_/*λ*_em_ = 379/469 nm). Furthermore, its peak extinction coefficient of 37,300 M^−1^ cm^−1^ and quantum yield of 0.13, are greater than those of split EBFP2 (Supplementary Table 1). When associated with GFP_11_-tagged β-actin or H2B, Capri_1–10_ exhibits very bright fluorescence in HeLa cells (Fig. 1c, d). To assess the improvement in the resulting brightness, we co-expressed GFP_11_-H2B in HEK 293T cells with either EBFP2_1-10_ or Capri_1-10_. Quantifying the fluorescence intensity of cells by flow cytometry, we found that Capri_1-10/11_ had a four-fold brighter fluorescence than EBFP2_1-10/11_ (Supplementary Fig. 4).

In addition to BFP variants, cyan-colored FPs have been widely studied. When we introduced substitutions into GFP_1-10_ (Y66W to make CFP_1-10_), complementation fluorescence was observed for GFP_11_-β-actin or GFP_11_-H2B fusions co-expressing with CFP_1-10_ in HeLa cells (Supplementary Fig. 5). However, it is noticeable in the figure that the overall brightness of CFP_1-10_ is relatively weak, making it difficult to visualize thin actin filaments (A recent in vitro assessment also reported that split CFP has a low brightness^10^). Therefore, we sought to produce a cyan-colored FP that has enhanced brightness. A recent improvement of full-length CFP, named Cerulean, increases the brightness by ~1.6 times^18^. Because the known substitutions are located only on GFP_1-10_ (Y66W/S72A/Y145A/H148D for Cerulean), Cerulean_1-10_ can associate with GFP_11_. To evaluate the enhancement in its overall brightness for cellular microscopy, we prepared plasmids encoding Cerulean_1-10_ or CFP_1-10_. We co-transfected each one of these plasmids with a GFP_11_-H2B plasmid in HEK 293T cells. Imaging by confocal microscopy, we quantified the signal level of these split FPs. We found that Cerulean_1-10_ signal was ~1.7 times brighter than that of CFP_1-10_ (*p* < 0.0001, Student’s *t*-test; Supplementary Fig. 6). We next assessed the performance of Cerulean_1-10_ when used as a fusion tag. We used GFP_11_-fused β-actin or H2B and co-expressed each one with Cerulean_1-10_ in HeLa cells (Fig. 1e, f). Although we observed complementation of Cerulean_1-10_ in the appropriate locations, some cells exhibited thicker actin bundles, which we have never seen in cells expressing a full-length Cerulean fusion (Fig. 1e and Supplementary Fig. 7). Because this artifact is common for dimeric or tetrameric FPs when they are targeted to two-dimensional structures^19,20^, we suspect that Cerulean_1-10/11_ is an oligomeric split FP. Nonetheless, an in-depth investigation is required to validate such a property in split Cerulean and under more various experimental conditions.

### Engineering of a red-colored split FP variant based on mRuby3

Although developmental efforts are ongoing to improve the brightness of split sfCherry^2,13,14^, having spectrally distinct split red FPs would foster the gross usefulness of FP_11_-tags. Since split sfCherry2 has a far-red shifted emission peak at 610 nm, we sought to explore the evolution of orange-red FPs such as mKO2, mRuby3, mApple, and mScarlet-I^15,21–23^ in *E. coli*. Following the previously established approach^13^, we inserted a 30 amino-acid spacer between the 10th and 11th β-strand of the four FPs. The long spacer insertion greatly diminished colony fluorescence of mKO2, mApple, and mScarlet-I, while colonies expressing spacer-inserted mRuby3 remained fluorescent (Supplementary Fig. 8). To improve the brightness of the spacer-inserted mRuby3, we mutagenized it using error-prone PCR and then transformed into *E. coli*; the three brightest candidates were pooled and subjected to another round. After three rounds, brightness of the best candidate revamped six-fold relative to that of spacer-inserted mRuby3 (Supplementary Fig. 9). We found seven substitutions in mRuby3_1-10_ (M15T/Q27H/T31I/V106I/S113C/R126S/A154V) and termed this variant split mRuby4 (*λ*_abs_/*λ*_em_ = 557/592 nm; see also Supplementary Fig. 3 for its absorbance spectrum). Compared to split mRuby3, we created a particularly bright variant that has a higher extinction coefficient and increased quantum yield (Supplementary Table 1).

**Fig. 2:**
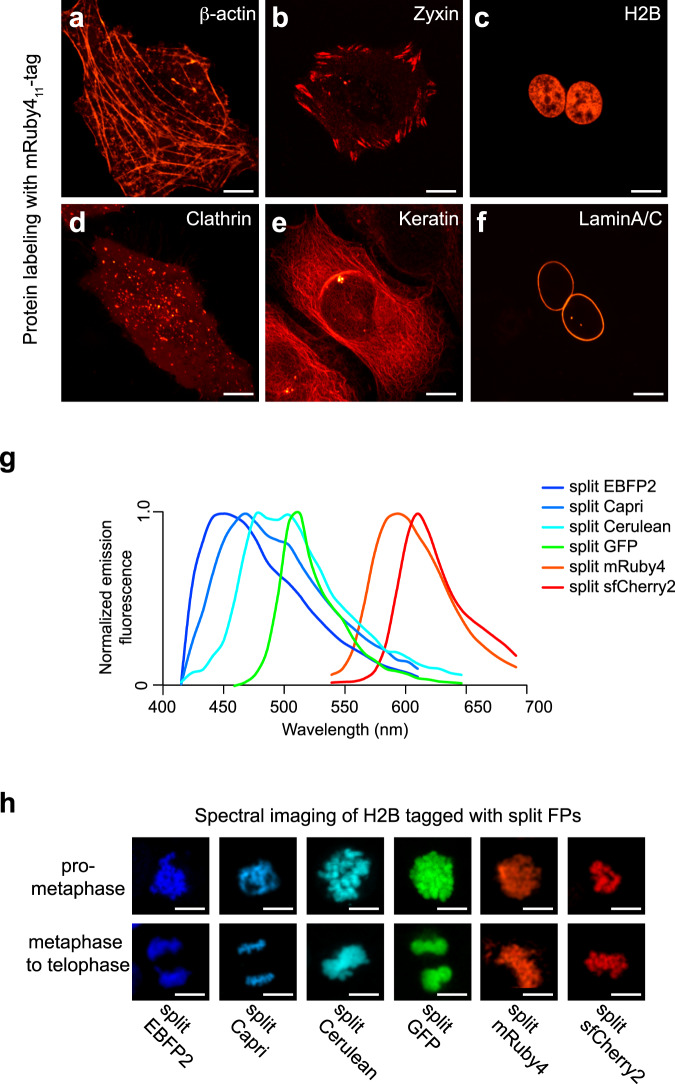
Development of mRuby4, a new red-colored split FP. **a**–**f** Cells co-expressing mRuby4_1-10_ with mRuby4_11_-fused cellular proteins. For each, the name of the fusion partner and its normal subcellular location are indicated, respectively; β-actin, actin stress fibers (**a**); Zyxin, focal adhesion (**b**); histone 2B, nuclei (**c**), Clathrin light chain, clathrin-coated pits (**d**); Keratin, intermediate filaments (**e**); Lamin A/C, nuclear envelops (**f**). **g** Normalized fluorescence emission spectra of FP_1-10/11_ variants in HeLa cells. (**h**) HEK 293 cells expressing H2B labeled with EBFP2_1-10/11_, Capri_1-10/11_, Cerulean_1-10/11_, GFP_1-10/11_, mRuby4_1-10/11_, or sfCherry2_1-10/11_ were co-cultured in the same plate. Spectrally unmixed images at the different stages of mitosis are represented (see also Supplementary Fig. 14). Scale bars,10 μm.

To assess whether split mRuby4 could fluoresce in human cells, we over-expressed mRuby4_11_-β-actin with mRuby4_1-10_ in HeLa cells. We observed that complemented split mRuby4 has a bright signal in fluorescent images of actin and various fusion proteins (Fig. 2a–f). To determine the signal level of split mRuby4, we performed a cellular fluorescence measurement by flow cytometry and compared the signal to full-length mRuby3. With expression of spacer-inserted mRuby4 in HEK 239T cells, we found that its signal level became around 69% of full-length mRuby3 (Supplementary Fig. 10). We have also demonstrated that mRuby4_1-10/11_ has sufficient efficiency to detect proteins expressed at endogenous levels. We employed CRISPR/Cas9-mediated HDR and introduced a 200-nucleotide ssDNA donor into the *HIST2H2BE* locus of HEK 293FT cells expressing mRuby4_1-10_. Subsequently, we found that split mRuby4 complementation had a prominent signal in images of the mRuby4_11_ knock-in (Supplementary Fig. 11).

As shown in Fig. 2g, the emission peaks for split mRuby4 and split sfCherry2 are only 20 nm apart yet still visually distinguishable (Supplementary Fig. 12). To further evaluate how many split FPs could be simultaneously visualized in different cells, we performed spectral imaging of HEK 293 cells expressing H2B fused proteins (Fig. 2g). We co-cultured six types of HEK 293 cells, each of which expressed H2B labeled with either EBFP2_1-10/11_, Capri_1-10/11_, Cerulean_1-10/11_, GFP_1-10/11_, mRuby4_1-10/11_, or sfCherry2_1-10/11_. After we synchronized at the G2/M phase by release from a cyclin-dependent kinase inhibitor, we imaged the cells (Supplementary Fig. 13). Within a couple of hours, 20% of the cell population was in cytokinesis, which is consistent with previous literature^24^. We then captured cells with each split FP fusion at different stages of mitosis (Fig. 2h). Overall, these experiments illustrate six-color spectral imaging of cellular proteins.

### Evaluation of split FP_1-10/11_ systems for multiplexed imaging in single cells

**Fig. 3:**
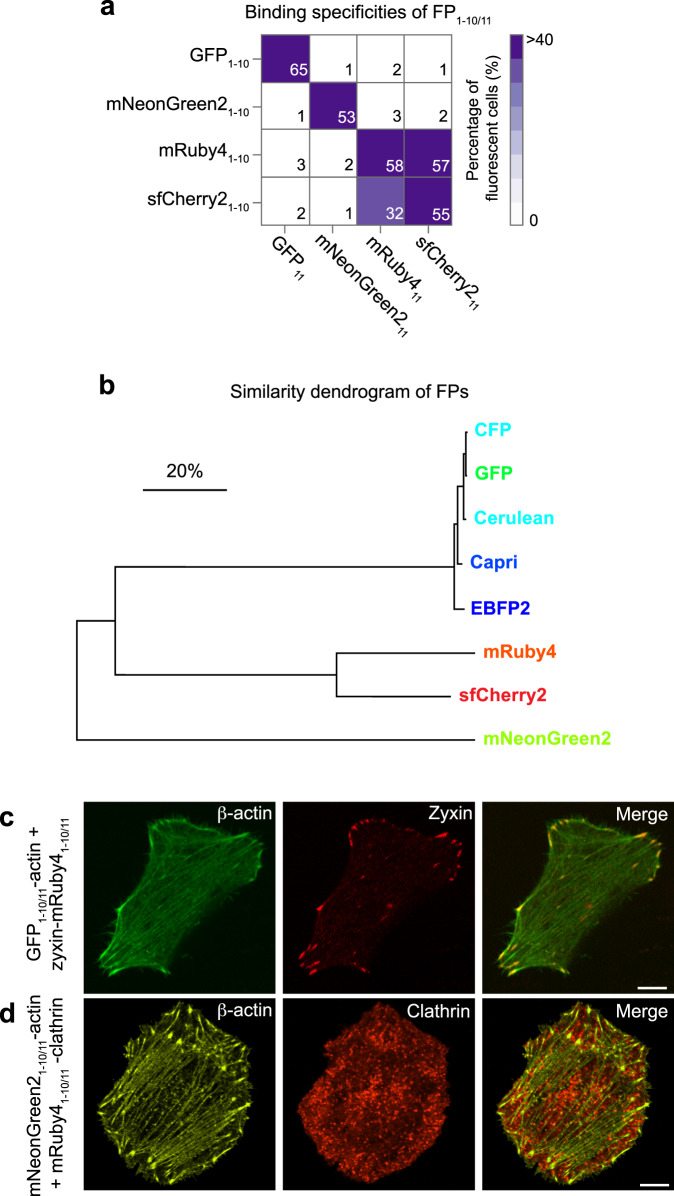
Characterizing the binding specificities of available FP_1-10/11_ pairs. **a** Characterizing the binding specificities of GFP_1-10/11_, sfCherry2_1-10/11_, mNeonGreen2_1-10/11_, and mRuby4_1-10/11_ by flow cytometry (see also Supplementary Fig. 15). Each of the FP_11_ fragments was tested for complementation to all of the FP_1-10_ fragments. Complementation is indicated as the percentage of fluorescent cells by a color scale and the number in each block. **b**, **c** Dual-color fluorescence images of HeLa cells expressing GFP_1-10/11_-β-actin and Zyxin-mRuby4_1-10/11_ (**b**), and mNeonGreen2_1-10/11_-β-actin and mRuby4_1-10/11_-Clathrin (**c**). **d** This dendrogram is based on the similarities of the following fluorescence protein sequences: EBFP2, Capri, Cerulean, CFP, GFP, mNeonGreen2, mRuby4, and sfCherry2. Proteins that share sequences are separated by smaller branch lengths. Scale bar, 20% dissimilarity. The dendrogram was constructed using MEGA 7.0 software. Scale bars, 10 μm.

An orthogonal interaction between GFP_1-10/11_ and sfCherry2_1-10/11_—meaning GFP_11_ can interact with GFP_1-10_, but cannot interact with sfCherry2_1-10_—is the basis of simultaneous labeling of two different fusion proteins in single cells. With an array of FP_1-10/11_ pairs developed, we sought to systematically test their binding specificities by flow cytometry. GFP_1-10/11_, sfCherry2_1-10/11_, mNeonGreen2_1-10/11_^13^, and mRuby4_1-10/11_ were examined for complementation in HEK 293T cells. Each FP_1-10_ fragment was co-expressed with any one of four FP_11_-fused β-actin, and the interactions were tested along the grid diagonal (Fig. 3a). As shown in Fig. 3a, all four FP_1-10_ fragments reconstituted with their corresponding partners. Interestingly, mRuby4_1-10_ and sfCherry2_11_ formed complementation signal as did mRuby4_11_ and sfCherry2_1-10_. Because the FP_1-10/11_ fragments encoded by closely related FPs, we expected there to be some crosstalk (Fig. 3b). We used HeLa cells co-expressing GFP_1-10/11_-β-actin with Zyxin-mRuby4_1-10/11_, or mNeonGreen2_1-10/11_-β-actin with mRuby4_1-10/11_-Clathrin to verify dual-color labeling with mRuby4_11_ in single cells. We found that the two distinct fluorescence channels did not overlap in the cells (Fig. 3c, d).

### A strategy to create new orthogonal split FPs using circularly permutated FP fragments

**Fig. 4:**
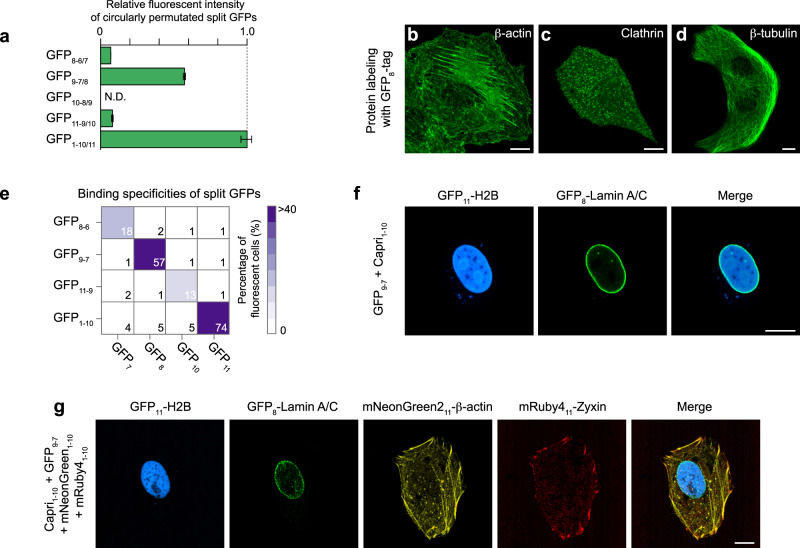
Multiplexed labeling of cellular proteins in human cells. **a** Fluorescence intensity of HEK 293T cells expressing actin labeled with circularly permutated split GFP variants, measured by flow cytometry (see also Supplementary Fig. 16). *n* = 698 cells for GFP_8-6/7_; *n* = 7792 for GFP_9-7/8_; *n* = 274 for GFP_11-9/10_; *n* = 11017 for GFP_1-10/11_. Error bars are SEM. **b**–**d** Confocal images of HeLa cells co-expressing GFP_9-7_ with GFP_8_ fusions; β-actin (**b**), Clathrin light chain (**c**), and β-tubulin (**d**). **e** The binding specificities of GFP_8-6/7_, GFP_9-7/8_, GFP_11-9/10_, and GFP_1-10/11_ were characterized by flow cytometry (see also Supplementary Fig. 17). **f** Dual-color fluorescence images of a U2OS cell expressing two different fusions, GFP_11_-H2B and GFP_8_-Lamin A/C. **g** Four-color images of a U2OS cell co-expressing GFP_11_-H2B, GFP_8_-LaminA/C, mNeonGreen2_11_-β-actin, and mRuby4_11_-Zyxin with GFP_1-10_, GFP_9-7_, mNeonGreen2_1-10_, and mRuby4_1-10_. Scale bars, 10 μm.

In order to provide more variants of split FPs orthogonal to existing FP_1-10/11_, we took advantage of circularly permutated GFP variants^25^. By linking the N-termini and C-termini and cutting out a single β-strand, any one of the eleven β-strands could be a split GFP-tag. We chose to measure complemented signal of the β-strands 7, 8, 9, and 10. (The β-strands 1–6 were excluded because the complementary fragments of these strands are unlikely to be water-soluble^26^). To this end, we prepared DNA constructs encoding each of the β-strands fused to β-actin and measured the overall complemented signal of each construct in HEK 293T cells by flow cytometry. We observed fluorescence signal reconstituted from the β-strands 7, 8, and 10 (hereafter named GFP_8-6/7_, GFP_9-7/8_, and GFP_11-9/10_) with their corresponding partners (Fig. 4a). These split GFPs retained 7–57% brightness of GFP_1-10/11_, albeit leaving room for improvement. To validate protein labeling using the β-strands, we generated constructs encoding various cellular proteins fused with GFP_8_ and co-expressed each one of them with GFP_9-7_ in HeLa cells. For three proteins tested, we observed their expected localizations (Fig. 4b–d).

Next, we assessed the binding specificities of GFP_8-6/7_, GFP_9-7/8_, GFP_11-9/10_, and GFP_1-10/11_. We performed the flow cytometry assay conducted in a grid format as described earlier. Either GFP_8-6_, GFP_9-7_, GFP_11-9_, or GFP_1-10_ was co-expressed with β-actin fused with the β-strands 7, 8, 9, or 11 in HEK 293T cells. In this experiment, each of the β-strands only binds to its corresponding partner (Fig. 4e). For instance, GFP_8_ interacts with GFP_9-7_, but not GFP_1-10_. This orthogonal interaction was validated by dual-color imaging of U2OS cells, in which GFP_11_-H2B and GFP_8_-Lamin A/C were co-expressed with Capri_1-10_ and GFP_9-7_. We observed the exclusion of GFP_8_-Lamin A/C from the nucleoplasm where GFP_11_-H2B predominately localized (Fig. 4f). Taken together, circularly permutated FP fragments can be used to generate additional orthogonal pairs for multiplexed split FP-labeling.

### Multicolor images reveal nuclear localization of Zyxin

Finally, we assessed the potential of split FP systems for multiplexed labeling of proteins in single cells. As a proof-of-principle, we used four orthogonal split FPs that we thoroughly investigated in this report (Capri_1-10/11_, GFP_9-7/8_, mNeonGreen2_1-10/11_, and mRuby4_1-10/11_), which are distinguishable from each other by using spectral imaging methodology (Fig. 4g). U2OS cells were transfected to express these split FPs targeted to four distinct proteins (H2B, Lamin A/C, β-actin, and Zyxin), and we observed their correct localization. Strikingly, a few cells displayed some portion of Zyxin proteins localized to the nucleus, although the proteins predominantly localized at focal adhesions in these cells. By visual inspection of a total of 145 cells, we found that 37 of these cells exhibited nuclear localization of Zyxin during interphase (Supplementary Fig. 18). Because Zyxin is a relatively large molecule (>80 kDa) but does not have a nuclear localization signal, Zyxin must enter the nucleus in contact with other components. We observed a similar nuclear localization pattern of Zyxin tagged with a full-length FP tag in U2OS cells (50 out of 174 cells), and found that this observation has also been confirmed in other cell lines^27–29^.

For the initial demonstration of multiplexed labeling, split FPs were over-expressed as fusions to target proteins in single cells (Fig. 4g). However, these fusion proteins might be subject to limitations because of the potential for overexpression artifacts (e.g., aberrant organelle and/or cellular morphology). To further verify our observation in the future, this approach will be extended to label endogenous proteins by methods such as CRISPR/Cas9-mediated gene knock-in^12^. Because a split FP tag is ~42–63 nucleotides long (which is ~10 times smaller than the size of an intact FP), a short donor oligo can be directly synthesized, making this a cloning free approach (see also Supplementary Fig. 11)^2,12^. In addition, a small tag such as GFP_11_-tag can be introduced into a host cell genome at high homologous recombination efficiencies^11^. Such a simple and efficient approach would facilitate the generation of multiple insertions in single cells. Thus, the sequences for multiple split FP tags could then be inserted either sequentially or simultaneously into targeted loci in individual cells stably expressing the complementary fragments, enabling multiplexed visualization of endogenous proteins.

## Methods

### Molecular cloning

The amino acid sequence of EBFP2 was obtained from a published report^16^ and slightly altered (see Supplementary Data 2). EBFP2 was split at the same site at GFP_1-10/11_. We introduced six substitutions to EBFP2_1-10_ (S65T/Q80R/F99S/V128T/M153T/V163A), which altered the EBFP2_1-10/11_ spectral property to Capri_1-10/11_. EBFP2_1-10_ and Capri_1-10_ were synthesized (Integrated DNA Technologies) and cloned into the mammalian expression vector pcDNA3.1 (Invitrogen) between KpnI and EcoRI (NEB) by using an In-Fusion HD cloning kit (Takara Bio). In order to clone specific DNA fragments into a plasmid vector, we used the In-Fusion HD cloning system in this entire study.

For the expression of CFP_1-10_ in mammalian cells, we introduced the corresponding point mutations into pcDNA3.1-GFP_1-10_ (Addgene #70219) using Q5 High-Fidelity DNA Polymerase (NEB). Primers used were as follows: CFP_1-10_-forward (5′-ACGCTTACGTggGGA GTTCAGTGC-3′), and CFP_1-10_-reverse (5′- TGTTACGAGAGTCGGCCA-3′). To express Cerulean_1-10_, we synthesized and cloned their DNA fragment into the KpnI/EcoRI sites of pcDNA3.1. For the nucleotide sequence of *Cerulean*_*1-10*_, see Supplementary Data 1.

GFP permits circular permutation of the amino acid sequence^25^. By linking the N- and C-termini and cutting out a single β-strand, any one of the eleven β-strands would become a new split GFP-tag. Huang et al.^26^ previously investigated all possible β-strands, and measured the solubility and relative reconstituted fluorescence intensity of each split GFP construct in *E. coli*. We tested Huang’s design of the β-strands 7, 8, 9, or 10 system in human cells. We constructed plasmids encoding each of the β-strands fused to β-actin. We used mEmerald-Actin-C-18 (Addgene #53978) as the template for PCR amplification of *ACTB*. The *ACTB* gene was amplified using primers, in which DNA sequences encoding the β-strands were included in part (For the sequence information of the primers, see Supplementary Data 3). The resultant PCR products were cloned into the KpnI/EcoRI sites of pcDNA3.1.

*GFP*_*8-6*_*, GFP*_*9-7*_*, GFP*_*10-8*_*, and GFP*_*11-9*_ were amplified from super-folder GFP OPT^1^ by PCR and inserted into the KpnI/EcoRI sites of pcDNA3.1. For the nucleotide sequences of *GFP*_*8-6*_*, GFP*_*9-7*_*, and GFP*_*11-9*_, see Supplementary Data 1.

To demonstrate the usefulness of mRuby4_11_, sfCherry2_11_, mNeonGreen2_11_, and GFP_8_, we labeled several cellular proteins with these split FP-tags. To construct plasmids of split FP-tag fusions, DNA fragments encoding cellular proteins were amplified by PCR with sets of primers (see also Supplementary Data 3) and cloned into the KpnI/EcoRI sites of pcDNA3.1. For the PCR amplification, we used the following DNA templates: mEmerald-Actin-C-18; mEmerald-Clathrin-15 (Addgene #54040); sfGFP-H2B-C-10 (Addgene #56367); pBABE-puro-GFP-wt-lamin A (Addgene #17662); mEmerald-Keratin-17 (Addgene #54134); sfGFP-Zyxin-6 (Addgene #56491).

The amino acid sequences of split FP-tags (i.e., GFP_7_, GFP_8_, GFP_10_, GFP_11_, mNeonGreen2_11_, sfCherry2_11_, and mRuby4_11_) were listed in Supplementary Table 2.

sfCherry2_1-10_, mNeonGreen2_1-10_, and sfGFP-Zyxin plasmids^13^ that can be transfected in mammalian cells are available through Addgene (#82603, #82610, and #56491, respectively).

### Mutagenesis and screening of libraries

When engineering split orange-red FP variants, we adopted a complementation assay previously described to optimize split mCherry2 in *E. coli*^13^. We inserted a 30-aa spacer (GGGGSEGGGSGGPGSGGEGSAGGGSAGGGS) between the tenth and eleventh β strands of each of the following fluorescent proteins; mKO2, mRuby3, mApple, and mScarlet-I^15,21–23^. The corresponding DNA sequences were directly synthesized and then cloned into the BamHI/XhoI sites of the *E. coli* expression vector pET28a (Novagen).

The longer spacer insertion eliminated colony fluorescence of mKO2, mApple, and mScarlet-I, whereas colonies expressing spacer-inserted mRuby3 gave low signal (Supplementary Fig. 8). To improve the brightness of spacer-inserted mRuby3, we mutagenized it by using a GeneMorph II Random Mutagenesis Kit (Agilent). Mutants were expressed and screened in pET28a. Plasmids were transformed into *E. cloni* EXPRESS Electrocompetent Cells (Lucigen). Transformation was performed by the Gene Pulser Electroporation Systems (BioRad). Colonies were grown on LB agar media (30 μg/mL Kanamycin) at 37 °C for 24 h and for additional 12–48 h at 37 °C after induction with 1 mM IPTG. For each round of mutagenesis, the number of colonies screened was at least 1 × 10^4^. Colonies expressing spacer-inserted mRuby3 variants were screened for fluorescence with the ChemiDoc Imaging System (BioRad). The imaging system was equipped with an Epi-green 520–545 nm excitation source, a Green Epi 605/50 filter, and a cooled CCD camera.

Through library screening, we obtained an extremely bright variant of spacer-inserted mRuby3, which we named spacer-inserted mRuby4 (Supplementary Fig. 9). The *mRuby4*_*1-10*_ sequence of spacer-inserted mRuby4 was amplified by PCR and cloned into the KpnI/EcoRI sites of pcDNA3.1. For the nucleotide sequence of *mRuby4*_*1-10*_, see Supplementary Data 1.

### Protein production and characterization of FPs

For spectral characterization of FPs, we produced and purified recombinant proteins: full-length EBFP2, spacer-inserted EBFP2, spacer-inserted Capri, full-length mRuby3, spacer-inserted mRuby3, and spacer-inserted mRuby4 (the amino acid sequences of those were listed in Supplementary Data 2). We designed pET plasmids such that recombinant proteins were labeled at the C termini with poly-histidine tags. The plasmids were introduced into BL21(DE3) Competent *E. coli* cells (NEB) via transformation. Cells were grown in 250 mL LB medium at 37 °C for 6 h (OD_600_ = 0.5), induced with IPTG (1 mM) for 4 h, and harvested by centrifugation. Cell pellets were lysed by French press. His-tagged proteins were purified with HisPur Cobalt Resin (Pierce). Proteins were further desalted into PBS pH7.4 using a GE Healthcare illustra NAP column (GE Healthcare). Extinction coefficients were calculated using Beer-Lambert law^10^. Quantum yields were determined using EBFP2^16^, and Rhodamine B (Wako) as reference fluorophores. The absorbance signals of samples and reference were measured using a microreader (Biotek Synergy 2). Diluted samples and reference were added into a quartz fluorescence cuvette (Thorlabs), and their integrated fluorescence intensities were measured by a fluorescence spectrophotometer (Hitachi F-7100). With the quantum yield of reference to be known, the final quantum yields of samples were attained using:

$${\rm{Qs}} = {\rm{Qr}} \times ({\rm{Ar}}/{\rm{As}}) \times ({\rm{Es}}/{\rm{Er}}) \times ({\rm{ns}}/{\rm{nr}})^2\;[ r\,{\mathrm{and}} \, s \,{\mathrm{refer}}\,{\mathrm{to}}\,{\mathrm{the}}\,{\mathrm{reference}}\,{\mathrm{and}}\,{\mathrm{samples}}],$$

where *Q* is the quantum yield, *n* is the refractive index, *A* is the absorbance of solution, and *E* is the integrated fluorescence intensity of emitted light.

### Fluorescence imaging

Confocal microscopy images of mRuby4_1-10/11_, GFP_1-10/11_ and mNeonGreen2_1-10/11_ were acquired on an inverted fluorescence microscope (Ti-E, Nikon) with a 100 × 1.45 NA oil immersion objective (Plan Apo, Nikon). The microscope was attached to the Dragonfly Spinning disk confocal unit (CR-DFLY-501, Andor). Two excitation lasers (40 mW 488 nm and 50 mW 561 nm lasers) were coupled to a multimode fiber passing through the Andor Borealis unit. A dichroic mirror (Dragonfly laser dichroic for 405-488-561-640) and band-pass filters (525/50 nm and 600/50 nm bandpass emission wheel filters) were selected for two-color imaging. The images were recorded with an EM-CCD camera (iXon, Andor).

Confocal microscopy images of EBFP2_1-10/11_, Capri_1-10/11_, CFP_1-10/11_, and Cerulean_1-10/11_, were collected by using an upright microscope (Axio imager Z2, Zeiss) with a 63 × 1.4 NA oil immersion objective (Plan Apo, Zeiss). The upright microscope had the LSM 880 Scan-head (Zeiss) with 32 channel GaAsP spectral PMT detector. It was equipped with six laser lines (Diode 405 nm; Argon 458, 488, 514 nm; HeNe 543, 633 nm). We used the 405-nm diode laser for EBFP2_1-10/11_, and Capri_1-10/11_ (main beam filter MBS-405 and 488/543, 409–491 nm barrier filter), the 458-nm Argon line for CFP_1-10/11_, and Cerulean_1-10/11_ (main beam filter MBS-458/514, 454–518 nm barrier filter). To characterize a spectrum from each individual FP, spectral images were acquired. We used 8.9 nm channel widths, meaning that each of the 32 channels on the spectral detector captured light over 8.9 nm bandwidth of the visible and near infrared spectrum. Sequential spectral image acquisitions were achieved in order of ascending wavelength of the excitation laser: 405, 488, and 543 nm. A typical data set consisted of 32 images (1024 × 1024 pixels), corresponding to different wavelengths from 410 to 695 nm. We then performed linear unmixing (Figs. 2h,  4g, and Supplementary Figs. 12–14) using Carl Zeiss’ Zen software.

Confocal microcopy images showed average intensity z projections, unless otherwise noted in the figure legends. Analysis of the confocal images was performed on Fiji software (NIH).

### Flow cytometry

Fluorescence of cells was measured using CytoFLEX (Beckman Coulter) in the CTEGD Cytometry Shared Resource at the University of Georgia (UGA). The instrument had four excitation lasers (405, 488, 561, and 610 nm) and three band-pass filters (450/45, 525/40, and 610/20 nm). Post-acquisition analysis was carried out using FlowJo software (Treestar, Inc).

### Cell culture, transfection, and drug treatment

HEK 293, HEK 293T, HeLa, and U2OS cells (gifted by Drs. Eggenschwiler and Kipreos, UGA) were grown in Dullbecco’s Eagle’s medium (HyClone), supplemented with fetal bovine serum (10%, *v/v*; Atlanta Biologicals) and penicillin/streptomycin (100 U/mL penicillin and 100 μg/mL streptomycin; HyClone). Cells were cultured at 37 °C and 5% CO_2_ in a humidified incubator. Plasmids were transfected at 400–800 ng DNA per well with Lipofectamine 2000 (3 μL, Invitrogen) or polyethylenimine (3 μL of 1 mg/mL PEI, Polysciences, Inc.) into Nunc Lab-Tek II Chambered Cover Glass (size: 8 wells, Nalge Nunc International) or Corning Costar Cell Culture Plates (size: 12 or 24 wells, Corning). In particular, for the quantitative comparisons in cellular fluorescence intensity (Fig. 4a and Supplementary Figs. 4 and 6), we transfected the plasmids of FP-tags and their partners at 400 ng and 800 ng, respectively. Cells were fixed with buffered 4% paraformaldehyde (Electron Microscope Sciences), mounted with PBS, and imaged by confocal microscopy.

For spectral imaging of multicolor H2B fusions (Fig. 2h and Supplementary Figs. 13 and 14), we used a Cdk1 inhibitor (10 μM of RO-3306, Sigma-Aldrich) to synchronize HEK 293 cells. HEK 293 cells were treated with the inhibitor for 18 h, blocked in the G2/M phase. For release from the inhibitor, we washed the culture five times with prewarmed culture media. Released cells returned to normal cell cycle progression, and were eventually fixed with 100% ice-cold methanol and mounted with PBS for microscopy.

### Knock-in cell creation

For knock-in of mRuby4_11_ into the *HIST2H2BE* locus, we ordered 200-nt HDR templates in single-stranded DNA (5′-gcccggcgagctggccaagcacgccgtgtccgagggcaccaaggcggtcaccaagtacaccagctccaagGGTGGCGGCGAAACCTACGTAGTGCAAAGAGAAGTGGCAGTTGCCAAATACAGCAACtgagtccctgccgggacctggcgctcgctcgctcgagtcgccggctgcttgactccaaaggctcttttcagag-3′, Integrated DNA Technologies). Cas9 protein was expressed in *E. coli* and purified by the Kipreos laboratory at UGA as described previously^11^. sgRNA and Cas9/sgRNA ribonucleoprotein complexes were prepared as described before^12^. After the treatment of HEK293 FT cells with nocodazole (200 ng/mL, Sigma-Aldrich) for 16 h, we performed electroporation on an Amaxa Nucleofector 2b device with Nucleofector Solution V reagents (Lonza).

Nocodazole-treated cells were resuspended at a concentration of 1 × 10^4^ cells/μL in 100 μL of Nucleofector Solution V. We added cells to the RNP/donor template mixture (50 μL), electroporated using the Q-001 program, and quickly transferred to 12-well plates with pre-warmed media. Electroporated cells were cultured for 2–5 days and transfected with mRuby4_1-10_ plasmid.

### Statistics and reproducibility

All experiments for the measurement of signal levels were replicated multiple times independently. Statistical analyses were performed using GraphPad Prism 7. Error bars in all figures refer to the standard error of the mean.

### Reporting summary

Further information on research design is available in the Nature Research Reporting Summary linked to this article.

## Supplementary information

Peer Review File

Supplementary Information

Description of Supplementary Files

Supplementary Data 1

Supplementary Data 2

Supplementary Data 3

Reporting Summary

## Data Availability

Relevant plasmids and sequences have been deposited in Addgene (www.addgene.org). The raw data referring to the plots shown in the main figures are provided in Supplementary figures. All relevant data are available from authors upon requests.

## References

[1] Cabantous S, Terwilliger TC, Waldo GS (2005). Protein tagging and detection with engineered self-assembling fragments of green fluorescent protein. Nat. Biotechnol..

[2] Kamiyama D (2016). Versatile protein tagging in cells with split fluorescent protein. Nat. Commun..

[3] Romei MG, Boxer SG (2019). Split green fluorescent proteins: scope, limitations, and outlook. Annu. Rev. Biophys..

[4] Pedelacq JD, Cabantous S (2019). Development and applications of superfolder and split fluorescent protein detection systems in biology. Int J. Mol. Sci..

[5] Pinaud F, Dahan M (2011). Targeting and imaging single biomolecules in living cells by complementation-activated light microscopy with split-fluorescent proteins. Proc. Natl Acad. Sci. USA.

[6] Koker T (2018). Cellular imaging by targeted assembly of hot-spot SERS and photoacoustic nanoprobes using split-fluorescent protein scaffolds. Nat. Commun..

[7] Kim YE, Kim YN, Kim JA, Kim HM, Jung Y (2015). Green fluorescent protein nanopolygons as monodisperse supramolecular assemblies of functional proteins with defined valency. Nat. Commun..

[8] Chun W, Waldo GS, Johnson GV, Split GFP (2011). complementation assay for quantitative measurement of tau aggregation in situ. Methods Mol. Biol..

[9] Van Engelenburg SB, Palmer AE (2010). Imaging type-III secretion reveals dynamics and spatial segregation of Salmonella effectors. Nat. Methods.

[10] Koker T, Fernandez A, Pinaud F (2018). Characterization of split fluorescent protein variants and quantitative analyses of their self-assembly process. Sci. Rep..

[11] Paix A (2017). Precision genome editing using synthesis-dependent repair of Cas9-induced DNA breaks. Proc. Natl Acad. Sci. USA.

[12] Leonetti MD, Sekine S, Kamiyama D, Weissman JS, Huang B (2016). A scalable strategy for high-throughput GFP tagging of endogenous human proteins. Proc. Natl Acad. Sci. USA.

[13] Feng S (2017). Improved split fluorescent proteins for endogenous protein labeling. Nat. Commun..

[14] Feng S (2019). Bright split red fluorescent proteins for the visualization of endogenous proteins and synapses. Commun. Biol..

[15] Bajar BT (2016). Improving brightness and photostability of green and red fluorescent proteins for live cell imaging and FRET reporting. Sci. Rep..

[16] Ai HW, Shaner NC, Cheng Z, Tsien RY, Campbell RE (2007). Exploration of new chromophore structures leads to the identification of improved blue fluorescent proteins. Biochemistry.

[17] Pedelacq JD, Cabantous S, Tran T, Terwilliger TC, Waldo GS (2006). Engineering and characterization of a superfolder green fluorescent protein. Nat. Biotechnol..

[18] Rizzo MA, Springer GH, Granada B, Piston DW (2004). An improved cyan fluorescent protein variant useful for FRET. Nat. Biotechnol..

[19] Day RN, Davidson MW (2009). The fluorescent protein palette: tools for cellular imaging. Chem. Soc. Rev..

[20] Cranfill PJ (2016). Quantitative assessment of fluorescent proteins. Nat. Methods.

[21] Sakaue-Sawano A (2008). Visualizing spatiotemporal dynamics of multicellular cell-cycle progression. Cell.

[22] Shaner NC (2008). Improving the photostability of bright monomeric orange and red fluorescent proteins. Nat. Methods.

[23] Bindels DS (2017). mScarlet: a bright monomeric red fluorescent protein for cellular imaging. Nat. Methods.

[24] Vassilev LT (2006). Selective small-molecule inhibitor reveals critical mitotic functions of human CDK1. Proc. Natl Acad. Sci. USA.

[25] Baird GS, Zacharias DA, Tsien RY (1999). Circular permutation and receptor insertion within green fluorescent proteins. Proc. Natl Acad. Sci. USA.

[26] Huang YM, Nayak S, Bystroff C (2011). Quantitative in vivo solubility and reconstitution of truncated circular permutants of green fluorescent protein. Protein Sci..

[27] Fujita Y (2009). Zyxin is a novel interacting partner for SIRT1. BMC Cell Biol..

[28] Cattaruzza M, Lattrich C, Hecker M (2004). Focal adhesion protein zyxin is a mechanosensitive modulator of gene expression in vascular smooth muscle cells. Hypertension.

[29] Hervy M, Hoffman LM, Jensen CC, Smith M, Beckerle MC (2010). The LIM protein zyxin binds CARP-1 and promotes apoptosis. Genes Cancer.

