# Supplementary material for: Multiplexed labeling of cellular proteins with split fluorescent protein tags
Source: Commun Biol. 2021 Feb 26;4:257. doi: 10.1038/s42003-021-01780-4 (PMC7910571; doi:10.1038/s42003-021-01780-4)
Supplement: Supplementary file 2 — Supplementary Information [file 42003_2021_1780_MOESM2_ESM.pdf]

Multiplexed labeling of cellular proteins with split fluorescent protein tags

Ryo Tamura, Fangchao Jiang, Jin Xie, and Daichi Kamiyama

Supplementary Figures 1 – 18

Supplementary Tables 1 – 2

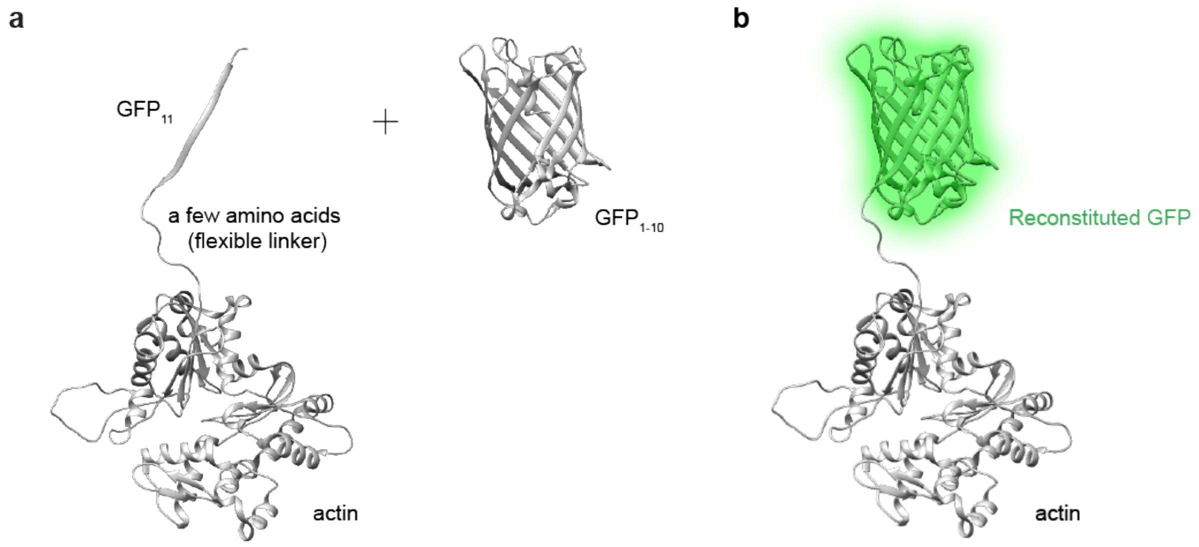

Supplementary Figure 1

Schematic diagram of GFP11-tag

Schematic of labeling proteins with split GFP is illustrated. A short region of split GFP (GFP_{11}) can be inserted into a gene of interest. The remainder ($GFP_{1\text{-}10}$), which is needed for reconstitution of GFP fluorescence, is expressed in cells. Importantly, $GFP_{1\text{-}10}$ does not produce background fluorescence. In this diagram, the N-terminus of β-actin is labeled with GFP_{11}-tag (**a**). The $GFP_{1\text{-}10}$ and GFP_{11} fragments can associate by themselves and form a functional GFP (**b**).

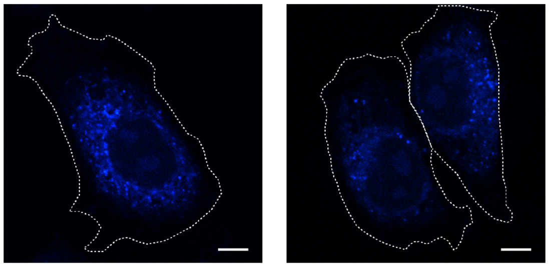

Supplementary Figure 2

Auto-fluorescence in cellular imaging with illumination at 405nm

Under 405 nm illumination, blue fluorescence can frequently be collected from auto-fluorescence in the perinuclear region. Representative images of un-transfected HeLa cells in this figure and **Figure 1** were taken with the same acquisition settings. Cells are outlined by white dash lines. Scale bar, 10 μm.

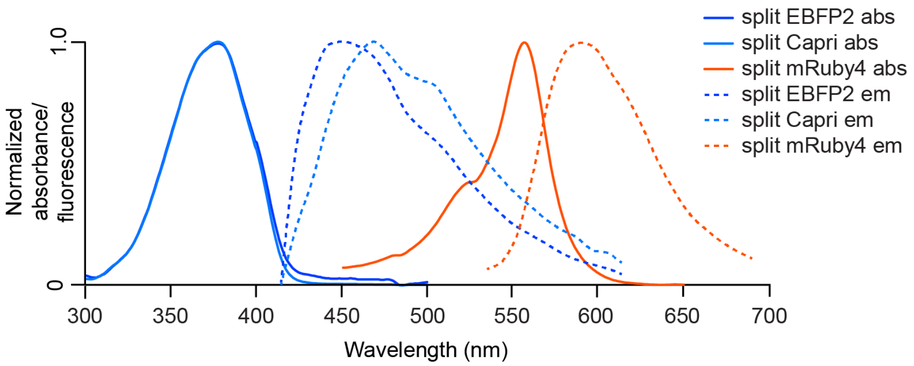

Supplementary Figure 3

Absorbance measurements and fluorescence emission of split EBFP2, split Capri, and split mRuby4

Normalized absorbance (solid lines) and emission (dotted lines) spectra of split EBFP2, split Capri, and split mRuby4.

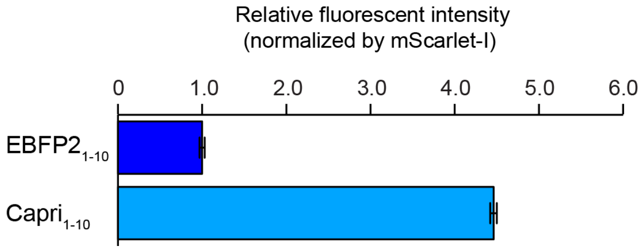

Supplementary Figure 4

Cellular fluorescence measurement of split BFP variants

Relative fluorescence intensity of HEK 293T cells expressing H2B labeled with $EBFP2_{1-10/11}$ or $Capri_{1-10/11}$, measured by flow cytometry and normalized to mScarlet-I signal. $EBFP2_{1-10}$ or $Capri_{1-10}$ was directly fused to mScarlet-I, of which the signal was applied to normalize the differences of gene expression levels. n = 13005 cells for $EBFP2_{1-10}$; n = 15987 cells for $Capri_{1-10}$. Error bars are SEM (Standard error of the mean).

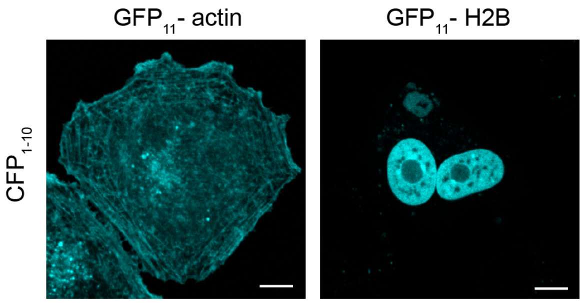

Supplementary Figure 5

Confocal microscopy images of cellular proteins labeled with split CFP

Fluorescence images of HeLa cells expressing either β-actin or histone 2B fusion of $CFP_{1\text{-}10/11}$. Scale bars, 10 μm.

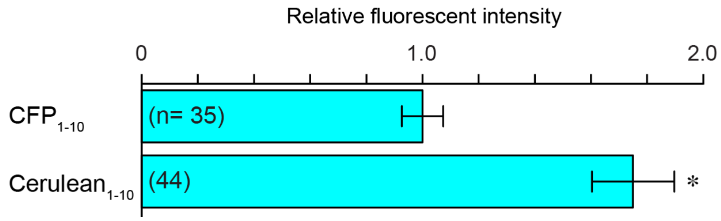

Supplementary Figure 6

Cellular fluorescence measurement of split CFP and split Cerulean

Relative fluorescence intensity of HEK 293T cells co-expressing GFP_{11}-H2B with $Cerulean_{1-10}$ or CFP_{1-10}, measured by confocal microscopy. After a background correction was applied, a fluorescent intensity value was measured as the sum intensity value of the nucleus for each cell. n = 35 - 44 cells. Error bars are SEM, $^{*}P$ = 0.0001 (*Student's t-tests*).

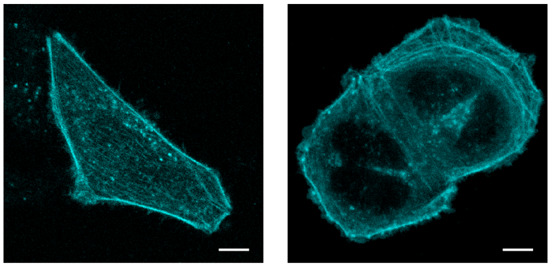

Supplementary Figure 7

Full-length Cerulean-β-actin expressed in HeLa cells

Representative confocal images of full-length Cerulean-targeted actin stress fibers in HeLa cells. Scale bars, 10 μm.

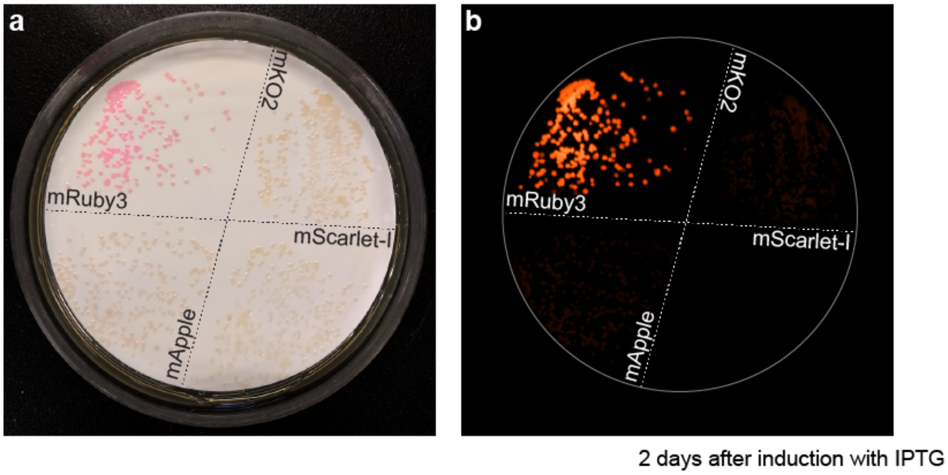

Supplementary Figure 8

Colony fluorescence measurement of spacer-inserted orange-red FPs

Transformed colonies of *E. coli* with four different constructs of spacer-inserted FPs (i.e., mKO2, mRuby3, mApple, and mScarlet-I) are shown in visible light (**a**) and 520 nm light (**b**). After induction with IPTG, the plate was incubated at 37 °C for 2 days.

a

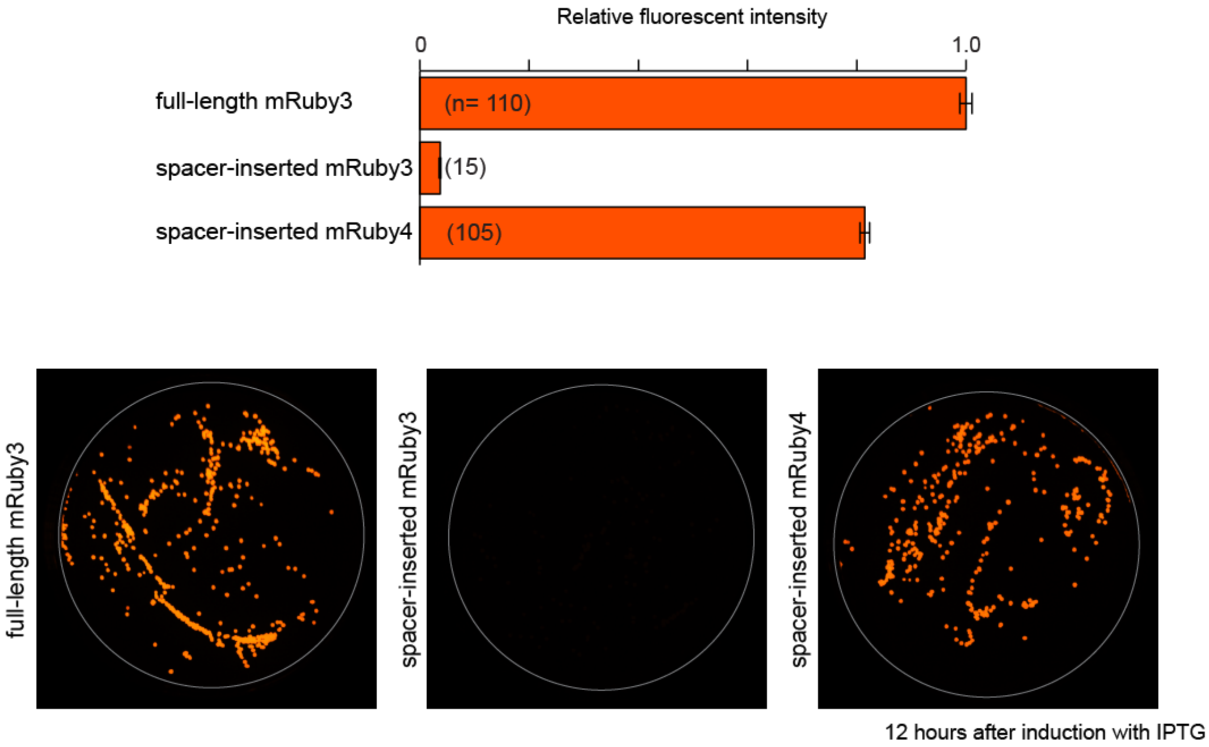

Supplementary Figure 9

Engineering the self-complementing split mRuby system in *E. coli*

(**a**) Relative fluorescence intensity of *E. coli* colonies expressing full-length mRuby3, spacer-inserted mRuby3, or spacer-inserted mRuby4. n = 15 - 110 colonies. Error bars are SEM. (**b**) Representative fluorescent images of *E. coli* colonies expressing full-length mRuby3, spacer-inserted mRuby3, or spacer-inserted mRuby4. These plates were kept at 37 °C for 12 hours after induction with IPTG.

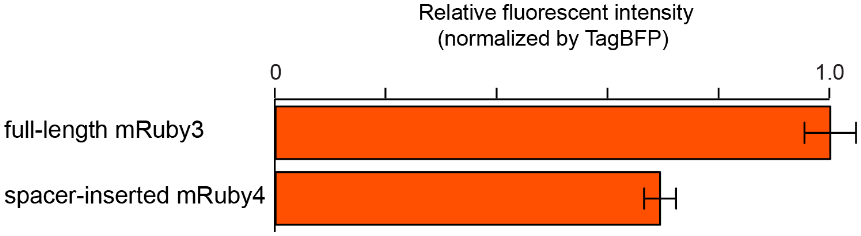

Supplementary Figure 10

Cellular fluorescence measurement of full-length mRuby3 and spacer-inserted mRuby4 in HEK cells

Relative fluorescence intensity of HEK 293T cells expressing actin labeled with two different FPs (full-length mRuby3, or spacer-inserted mRuby4), measured by flow cytometry. Full-length mRuby3, and spacer inserted-mRuby4 were directly fused to TagBFP. TagBFP signal was used to normalize gene expression levels. n = 6079 cells for full-length mRuby3; n = 10051 cells for spacer-inserted mRuby4. Error bars are SEM.

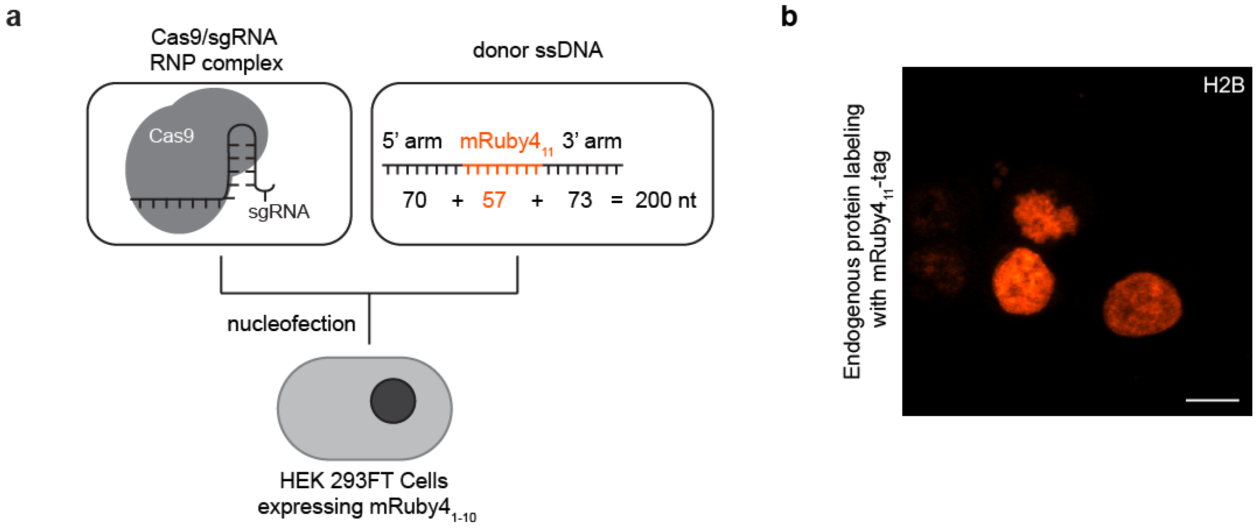

Supplementary Figure 11

mRuby4$_{11}$ labeling of endogenous proteins

(**a**) Schematic diagram of endogenous protein labeling with mRuby4$_{11}$. *mRuby4$_{11}$* is only 57 nt including a short amino-acid linker, and thus, we can adopt relatively short homology arms (70-73 nucleotides on both 5' and 3' arms). Donor DNA consequently becomes a 200-nucleotide single-strand DNA. (**b**) Fluorescent image of mRuby4$_{11}$ knock-in in HEK 293FT cells. We knocked mRuby4$_{11}$ into the H2B locus (*HIST2H2BE*). Cas9/sgRNA RNP nucleofection enabled us to obtain 0.5 % of mRuby4$_{1\text{-}10/11}$-positive cells. Scale bars, 10 μm.

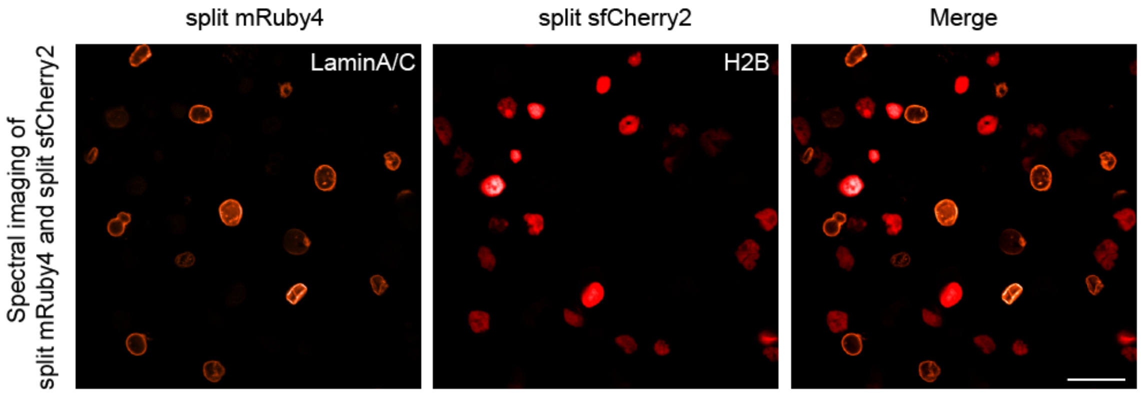

Supplementary Figure 12

Distinguishing split sfCherry2 and split mRuby4 by their emission spectra

HEK 293T cells expressing either sfCherry2$_{1\text{-}10/11}$-H2B or mRuby4$_{1\text{-}10/11}$-LaminA/C were co-cultured in the same plate. Two fluorescent images were acquired using confocal microscopy with spectral detection. Emission was detected from 463 to 695 nm using excitation of 514-nm and 543-nm laser. Unmixed channels are overlaid and shown in pseudo-colors. Scale bars, 25 μm.

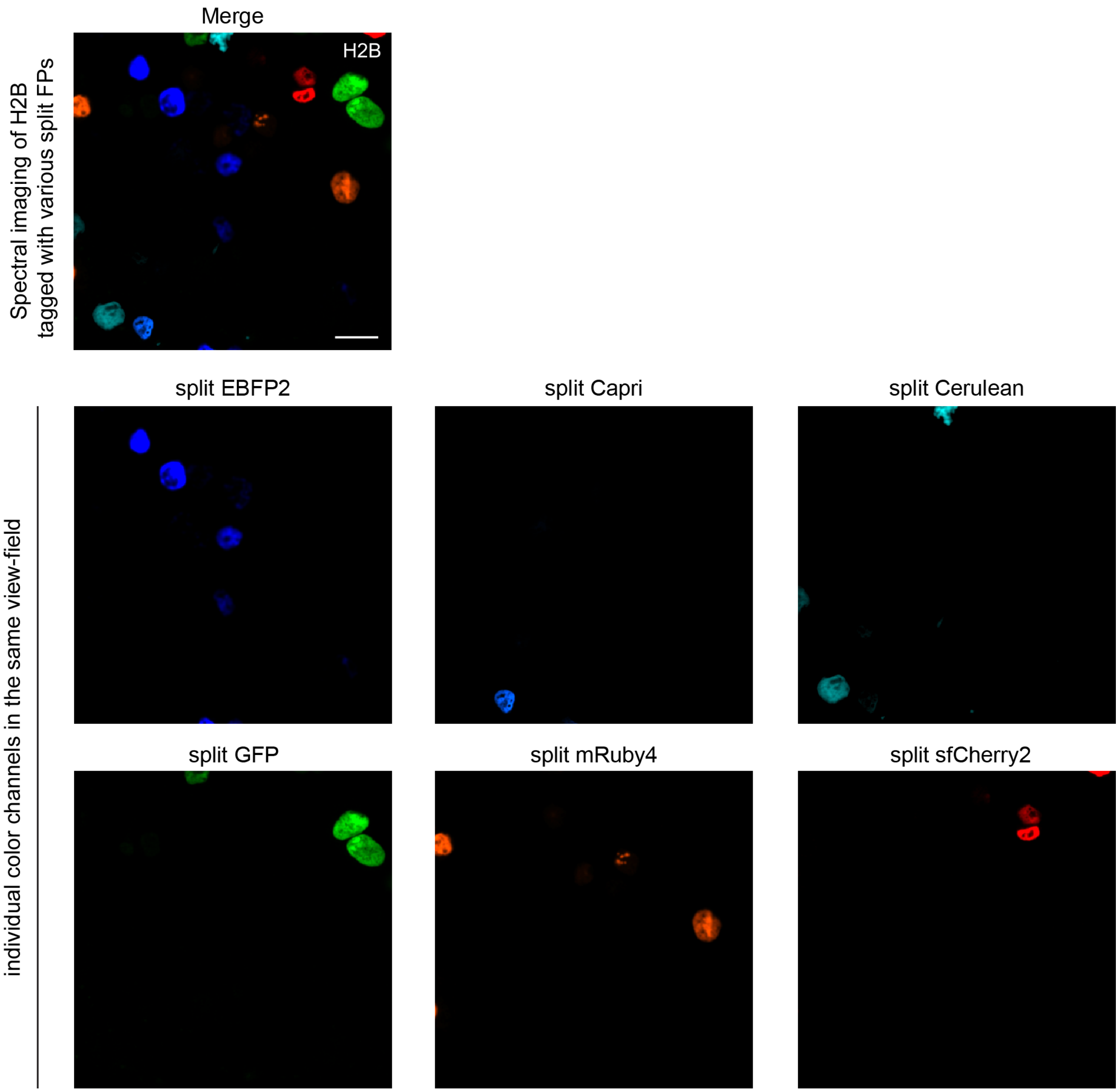

Supplementary Figure 13

Spectral imaging of H2B fusions with multicolor split FPs

Representative images of HEK 293 cells expressing H2B labeled with either $EBFP2_{1\text{-}10/11}$, $Capri_{1\text{-}10/11}$, $Cerulean_{1\text{-}10/11}$, $GFP_{1\text{-}10/11}$, $mRuby4_{1\text{-}10/11}$, or $sfCherry2_{1\text{-}10/11}$. We collected images of the same view-field captured at emission wavelengths from 410 to 695 nm and subsequently obtained spectrally unmixed images of the six split FPs (see also the Methods). Scale bars, 25 μm.

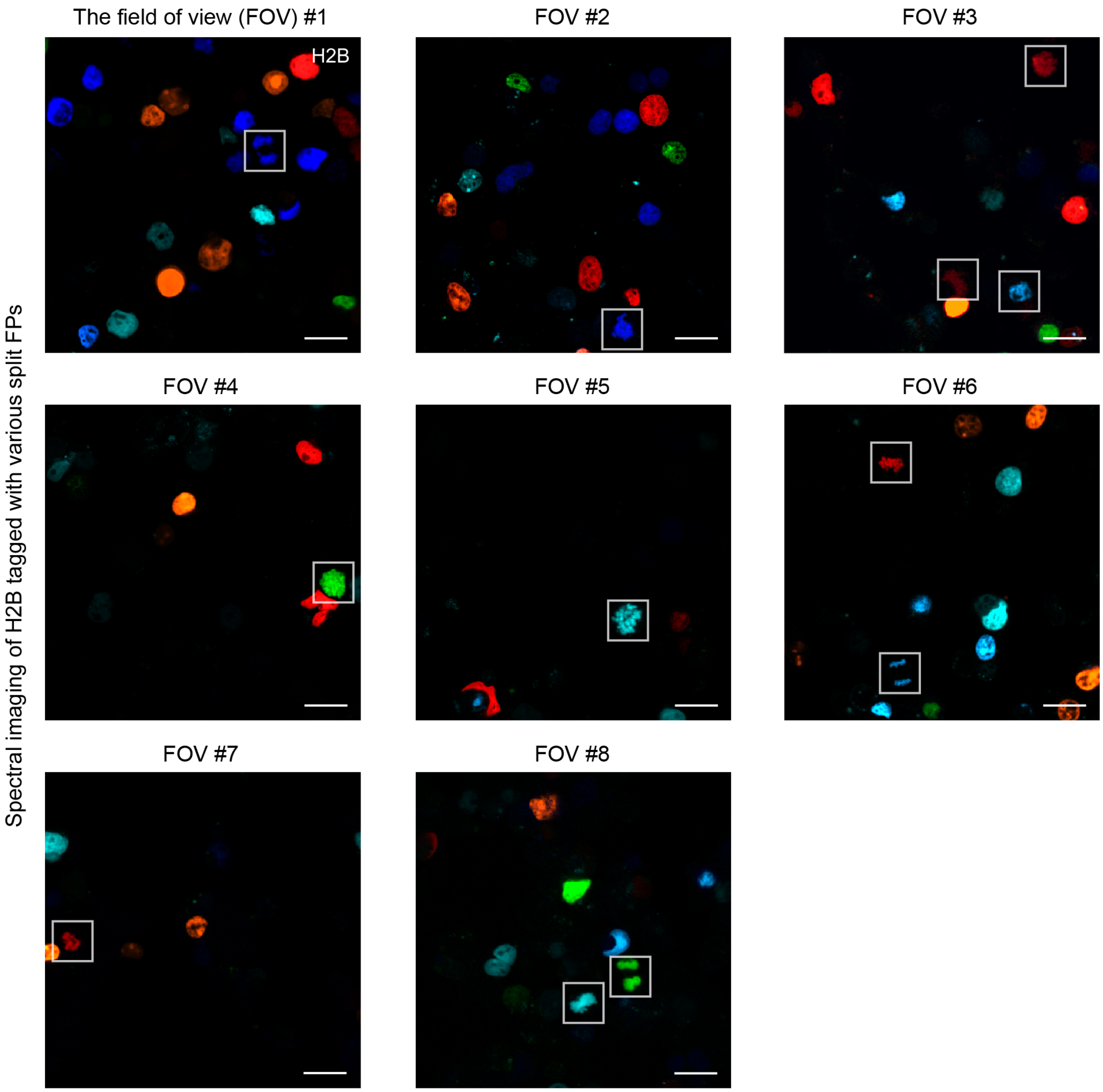

Supplementary Figure 14

Unprocessed images shown in Figure 2h

Original images used in Figure 2h. Unmixed channels are overlaid and shown in pseudo-colors. White boxes mark the regions in the main figure. This experiment was repeated three times with similar results. Scale bars, 25 μm.

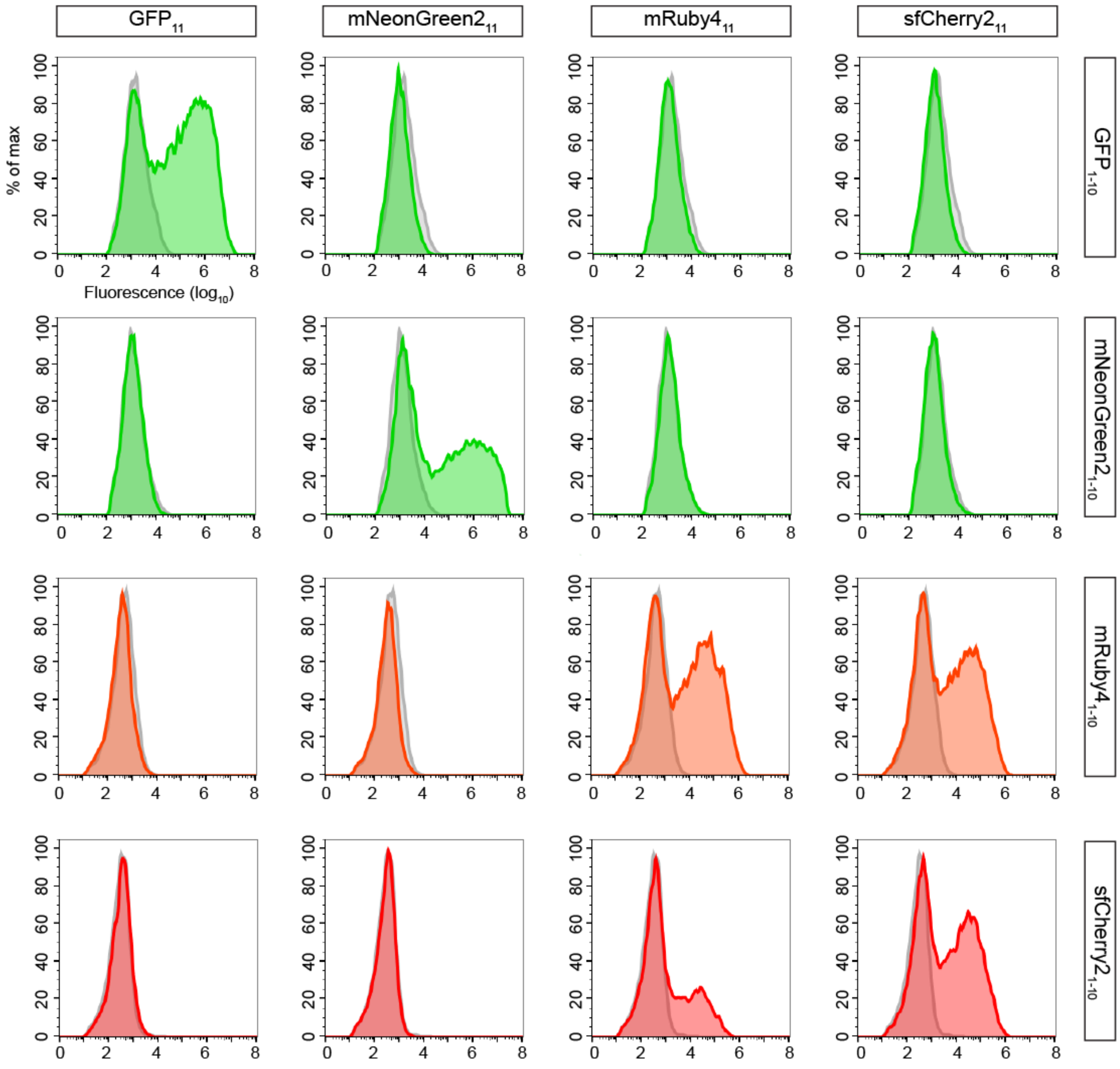

Supplementary Figure 15

Testing the binding specificities of $GFP_{1\text{-}10/11}$, $sfCherry2_{1\text{-}10/11}$, $mNeonGreen2_{1\text{-}10/11}$, and $mRuby4_{1\text{-}10/11}$

Each of the FP_{11} fragments was tested for complementation to all of the $FP_{1\text{-}10}$ fragments. The complemented signal was measured by flow cytometry. Samples were acquired using 488 nm or 561 nm excitation source. Measurements are represented as flow cytometry histograms. Overlay of a control population (HEK 293T cells transfected with $FP_{1\text{-}10}$ alone; gray) onto the split FP-expressing population (green, orange, and red) allows identification of the positive cells. A summary chart is shown in Figure 3a.

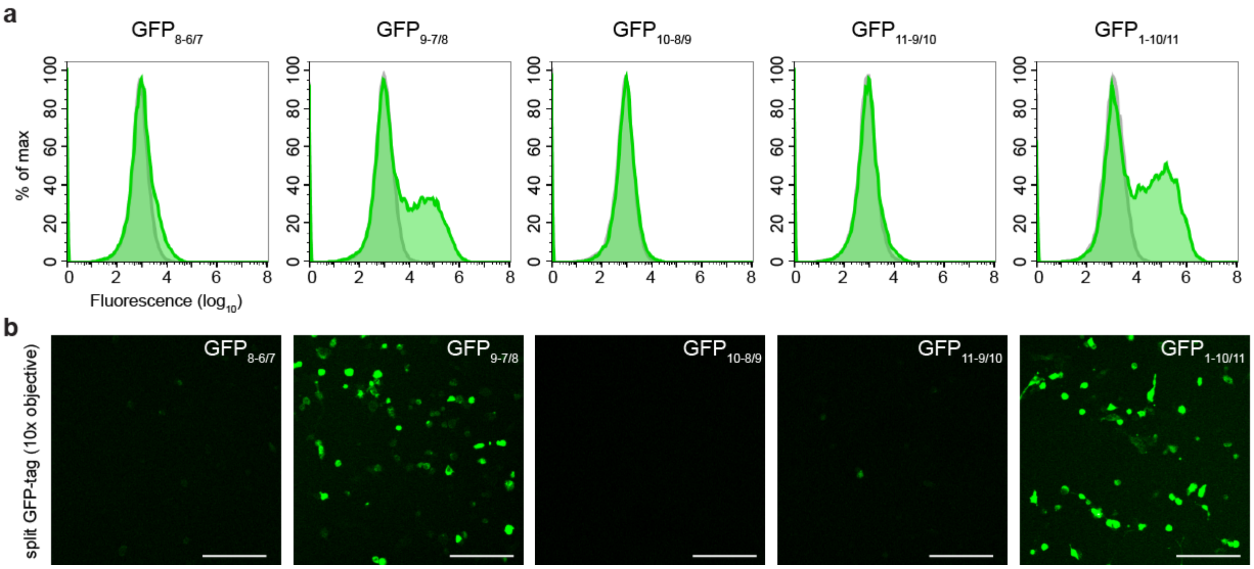

Supplementary Figure 16

Cellular fluorescence measurement of circularly permutated split GFP variants

(**a**) Fluorescence intensity of HEK 293T cells expressing actin labeled with $GFP_{8\text{-}6/7}$, $GFP_{9\text{-}7/8}$, $GFP_{10\text{-}8/9}$, $GFP_{11\text{-}9/10}$, and $GFP_{1\text{-}10/11}$, measured by flow cytometry (green). Control cells (HEK 293T cells transfected with either $GFP_{8\text{-}6}$, $GFP_{9\text{-}7}$, $GFP_{10\text{-}8}$, $GFP_{11\text{-}9}$ or $GFP_{1\text{-}10}$ alone) are represented in each histogram overlay (gray). A summary graph is shown in Figure 4a. (**b**) Their corresponding fluorescence images of HEK 293T cells. Fluorescence images were acquired with a 10x objective (Plan Fluor, Nikon). Scale bars are 200 μm.

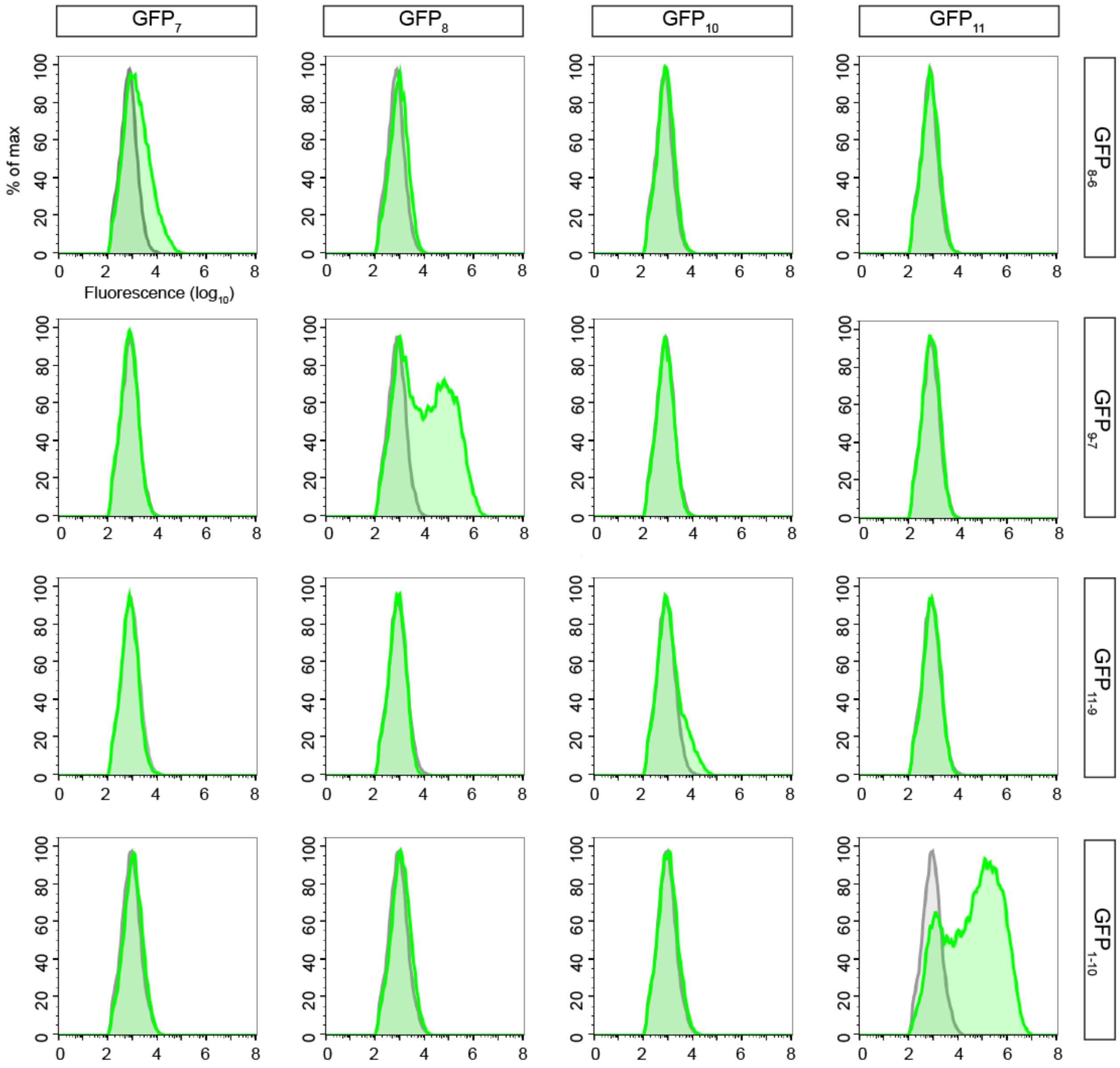

Supplementary Figure 17

Testing the binding specificities of $GFP_{8\text{-}6/7}$, $GFP_{9\text{-}7/8}$, $GFP_{11\text{-}9/10}$, and $GFP_{1\text{-}10/11}$

The β-strands 7, 8, 9, or 11 were tested for complementation to the $GFP_{8\text{-}6}$, $GFP_{9\text{-}7}$, $GFP_{11\text{-}9}$, or $GFP_{1\text{-}10}$ fragments. The complemented signal (green) was measured by flow cytometry using 488 nm excitation source. Control cells (HEK 293T cells transfected with either $GFP_{8\text{-}6}$, $GFP_{9\text{-}7}$, $GFP_{11\text{-}9}$ or $GFP_{1\text{-}10}$ alone) are represented in each histogram overlay (gray). A summary chart is shown in Figure 4e.

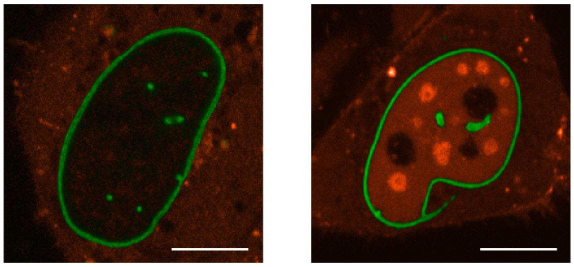

Supplementary Figure 18

Nuclear localization of zyxin

Representative single confocal sections of U2OS cells expressing $GFP_{9\text{-}7/8}$-LaminA/C (green) and $mRuby4_{1\text{-}10/11}$-Zyxin (red). While Zyxin mostly localized outside of the nucleus (left panel), some cells display the localization of Zyxin in the nucleoplasm (right panel). Scale bars, 10 μm.

Supplementary Table 1

Properties of split FPs engineered in this study

	EBFP2 [b]	split EBFP2	split Capri	mRuby3	split mRuby3	split mRuby4
**Absorbance peak (nm)**	378	379	379	556	557	557
**Emission peak (nm)**	450	450	469	593	593	592
**EC at peak ($M^{-1}cm^{-1}$)**	33,500	23,400	37,300	119,000	31,700	92,500
**QY**	0.09	0.07	0.13	0.48	0.23	0.32
**Brightness [a]**	3,015	1,638	4,849	57,120	7,291	29,600

EC, extinction coefficient; QY, quantum yield. [a] Calculated as the product of EC at peak and QY. [b] six substitutions (N40I/T106K/E112V/K166T/I167V/S206T) were introduced into the original EBFP2.

Supplementary Table 2

Amino acid sequences of split FP-tags.

GFP_7	KLEYNFNSHNVYITADKQ
GFP_8	KNGIKANFTVRHNV
GFP_{10}	LLPDNHYLSTQTVLSKDPNEK
$EBFP2_{11}$ $Capri_{11}$ CFP_{11} $Cerulean_{11}$ GFP_{11}	RDHMVLHEYVNAAGIT
$mNeonGreen2_{11}$	TELNFKEWQKAFTDMM
$mRuby4_{11}$ (∗)	ETYVVQREVAVAKYSN
$sfCherry2_{11}$	YTIVEQYERAEARHST

∗ The original 11th β-strand of $mRuby4_{11}$ consists of 26 amino acids (ETYVVQREVAVAKYSN**LGGGMDELYK**), and we removed the last 10 amino acids from it. These amino acids are unlikely to be essential because they form an unstructured polypeptide chain and do not provide the structural rigidity to the β-strand.
