# Supplementary material for: Multiplexed labeling of cellular proteins with split fluorescent protein tags
Source: Commun Biol. 2021 Feb 26;4:257. doi: 10.1038/s42003-021-01780-4 (PMC7910571; doi:10.1038/s42003-021-01780-4)
Supplement: Supplementary file 3 — Description of Supplementary Files [file 42003_2021_1780_MOESM3_ESM.pdf]

Description of Additional Supplementary Files

File name: Supplementary Data 1

Description: Nucleotide sequences of $EBFP2_{1-10}$, $Capri_{1-10}$, $Cerulean_{1-10}$, $mRuby4_{1-10}$, GFP_{8-6}, GFP_{9-7}, and GFP_{11-9}

File name: Supplementary Data 2

Description: Amino acid sequences of full-length EBFP2, spacer-inserted EBFP2, spacer-inserted Capri, full-length mRuby3, spacer-inserted mRuby3, and spacer-inserted mRuby4

File name: Supplementary Data 3

Description: List of primers used in this study
